# Comprehensive multi-omics and biochemical analysis to elucidate the molecular response mechanisms of gill and kidney tissues under acute salinity stress in *Pseudobagras ussuriensis*

**DOI:** 10.1186/s12864-025-11773-w

**Published:** 2025-07-01

**Authors:** Yu Liu, Libo Gu, Jun Zhao, Mengzhu Liu, Ke Wang, Qingbo Zhou, Yang Cao, Ruyi Hu, Weiwei Wang, Qing Liu

**Affiliations:** 1 College of Animal Science, Shanxi Agricultural University, Jinzhong, 030801 China; 2 Shanxi Key Laboratory of Animal Genetics Resource Utilization and Breeding, Jinzhong, 030801 China

**Keywords:** *Pseudobagras ussuriensis*, Salinity stress, Transcriptomics, Metabolomics, Oxidative enzyme activity

## Abstract

Acute salinity stress critically impacts aquaculture efficiency by inducing physiological disruptions in fish. This study investigated the molecular adaptation mechanisms of *Pseudobagrus ussuriensis* exposed to 10 ppt NaCl for 96 h, integrating transcriptomic and metabolomic analyses of gill and kidney tissues. In transcriptomics, 2,554 and 1,066 differentially expressed genes (DEGs) were identified in gills and kidneys, respectively, with significant enrichment in pathways related to energy metabolism (glycolysis, oxidative phosphorylation), membrane dynamics (glycerophospholipid metabolism), and immune-osmoregulatory crosstalk (HIF-1, TNF, and Jak-STAT signaling). Metabolomics revealed 85 and 433 differential metabolites (DMs) in gills and kidneys, highlighting tyrosine metabolism, amino acid biosynthesis/degradation, and lipid remodeling (e.g., glycerophospholipids, sphingolipids). Multi-organ coordination was observed: gills prioritized short-term osmotic adaptation via membrane lipid reorganization but suffered oxidative damage due to sustained downregulation of *ALDH7 A1* and *AOX/HADHA*, triggering a “membrane injury–oxidative stress-ATP depletion” cycle. Conversely, kidneys-maintained energy homeostasis through purine/pyrimidine-TCA cycle reprogramming and autophagy-apoptosis balance. Critically, interorgan metabolic crosstalk-mediated by lipid mediators (prostaglandins, sphingolipids) and amino acid derivatives (branched-chain keto acids, glutamine)—orchestrated substrate shuttling (e.g., lactate for energy exchange) and systemic signaling, bridging local stress responses (gill ion regulation) with global metabolic adjustments (renal energy buffering). Notably, the levels of phosphatidylcholine (PC) and phosphatidylethanolamine (PE) in the kidney and phosphatidic acid (PA) and PC in the gills were significantly increased, while sphingomyelin (SM) decreased. Our findings demonstrate that acute salinity stress induces organ-specific metabolic reprogramming and interorgan crosstalk in *P*. *ussuriensis*, revealing a trade-off between osmotic adaptation and oxidative stress resilience mediated by lipid remodeling and energy metabolism dysregulation.

**Supplementary Information:**

The online version contains supplementary material available at 10.1186/s12864-025-11773-w.

## Introduction

Salinity is closely related to the growth, development, and regulation of various physiological functions in fish. Changes in salinity force the fish to adapt to a series of physiological changes. Salinity is a key factor in artificial aquaculture conditions [1]. Fluctuations in salinity affect aquaculture on multiple levels, including biological immune responses, physiological adaptation mechanisms, and ecosystem stability.

Salinity stress disrupts immunological defenses and homeostatic regulation in commercially important aquatic species. For instance, Experimental evidence demonstrates that hypotonic stress (0–10‰ salinity) significantly impairs the immune competence of *Epinephelus coioides*, manifesting as suppressed cytokine expression (IL-1β, IL-6, IL-10, TNF-α), attenuated immune cell proliferation, and diminished inflammatory responses [2]. Similarly, acute salinity reduction below 10‰ critically compromises bivalve physiology, as evidenced in *Mytilus galloprovincialis* by complete cessation of byssal thread production, structural degeneration of pedal secretory glands, and ultimately fatal loss of adhesive capacity [3]. Salinity effects on the survival and metabolism of aquatic animals. For example, Under salinity stress, the survival rate and digestive enzyme activities (e.g., amylase and lipase) of *Perna viridis* were regulated [4]. The gill tissue of *Crassostrea hongkongensis* counteracts oxidative damage by modulating antioxidant enzyme activities and protein carbonylation levels [5]. The gill tissue of *Penaeus vannamei* exhibits adaptive structural and functional adjustments, including thickening of epithelial cells and enrichment of mitochondria to support active ion transport [6].

At the ecosystem level, salinity fluctuations may undermine the stability of aquaculture systems by affecting biodiversity. Studies have found that the diversity of estuarine fish communities can buffer the impacts of salinity changes via a “portfolio effect” (akin to risk diversification in investment portfolios), where asynchronous responses among different fish species help maintain overall productivity [1, 7, 8]. Additionally, nitrifying bacterial communities that have been pre-adapted to salinity stress (e.g., through pre-exposure to seawater) exhibit enhanced tolerance to subsequent salinity fluctuations, providing potential strategies for optimizing aquaculture wastewater treatment systems [9].

The regulation of salinity within fish primarily occurs in the gills and kidneys, where ion absorption is enhanced in the gills and diluted urine is produced in the kidneys to compensate for ion loss [10, 11]. Salinity stress can cause structural changes in fish tissues, such as alterations in gill structure and necrosis in the liver and kidney tissues. The Qinghai Lake naked carp (*Gymnocypris przewalskii*), which inhabits the high-altitude saline-alkaline, hypoxic, and oligotrophic waters of Qinghai Lake, exhibits significant adaptive modifications in its gill arches and filaments: the number of gill rakers increases with a denser arrangement and elongation, while the gill filaments display a highly vascularized structure [12]. The glomerular structure collapses, collecting tubules shrink, and the filtration rate of the nephron decreases significantly, leading to an obvious suppression of kidney function. Magnesium and calcium concentrations in the intestines are higher than those in the plasma, but no significant changes have been observed in the tissue structure [13]. Studies on *Oreochromis niloticus*, under salinity stress, show an increase in mitochondria-rich cells in the gill tissue, vacuolization, degenerative changes, necrosis in the liver, and significant interstitial nephritis in the kidneys [14].

Salinity stress also alters the oxidative stress levels in fish cells. For example, salinity significantly affects the activities of superoxide dismutase (SOD), catalase (CAT), glutathione peroxidase (GPX), and malondialdehyde (MDA). In *Rachycentron canadum* juveniles, the specific activities of SOD, CAT, and GPX increased with decreasing salinity [15]. Both acute and chronic salinity stress lead to a gradual increase in the activities of SOD and CAT in the blood of *Sebastes schlegeli* as seawater salinity decreased [16]. In *Poecilia reticulata*, antioxidant enzyme activity increases significantly under acute salinity stress at different salinity levels [17].

Salinity stress also causes changes in fish energy metabolism, immune responses, signaling molecules, and related metabolic pathways. Transcriptomic analysis of *Lateolabrax maculatus* under salinity stress has revealed that salinity-regulated genes are related to ion transport proteins, energy metabolism, signal transduction, immune responses, and structural remodeling [18]. In *Cynoglossus semilaevis* (half-smooth tongue sole), liver transcriptomic studies have shown that lipid metabolism is crucial for salinity adaptation [19]. Transcriptomic analysis of *Rachycentron canadum* under different salinity conditions showed that the major enriched pathways included steroid biosynthesis, unsaturated fatty acid metabolism, glutathione metabolism, energy metabolism, osmoregulation, and immune responses, with salinity having the greatest effect on metabolism [20]. Studies on *Carassius auratus* (goldfish) using transcriptomic and metabolomic analyses of gill tissues have found that saline-alkali stress disrupts the antioxidant system in the gills, disturbs lipid metabolism, and induces cell apoptosis and immune responses [21].

*Pseudobagrus ussuriensis*, also known as the Ussuri catfish, is highly valued for its tender flesh and high economic value [22]. It is widely cultured in more than ten provinces in China. Research on *P. ussuriensis* breeding has mainly focused on artificial propagation, aquaculture mode improvements, sex determination [23], genetic analysis [24], and stress resistance breeding [25]. However, Research on salinity-tolerant breeding holds significant scientific and practical value in aquaculture, ecological conservation, and addressing climate change. research on *P. ussuriensis* salt-tolerant breeding is limited. In this study, we investigated the physiological and biochemical changes in the gill and kidney tissues of *P. ussuriensis* under acute salinity stress for 0, 6, 12, 24, 48, 72, and 96 h. We performed multi-omics analyses, including transcriptomics and non-targeted metabolomics, on the gill and kidney tissues after 24 h of salinity stress, to understand the molecular-level impacts of acute salinity stress on these tissues. This study provides valuable data for understanding the molecular regulatory mechanisms in the gill and kidney tissues of *P. ussuriensis* under salinity and offers insights for salt and alkali resistance breeding in this species.

## Materials and methods

### Ethical statement

This study was conducted in accordance with the principles of animal protection, livestock, and ethical standards, and was authorized by the Animal Ethics Committee of Shanxi Agricultural University (Approval No.SXAU-EAW-2022 F.YC.012017254).

### Salinity design and experimental fish sampling

Juvenile *P. ussuriensis* were obtained from the breeding base in Yongji City, Shanxi Province. The fish were transported alive in oxygen bags to the laboratory and housed in a static water pool for acclimatization. The juveniles *P. ussuriensis* (body length: 10.50 ± 3.09 cm, body weight: 16.76 ± 6.38 g) were acclimatized and subjected to salinity treatments at the aquaculture laboratory of Shanxi Agricultural University. The fish were maintained in aerated tap water at a temperature of 20–25 °C for two weeks, under a natural light cycle, with dissolved oxygen levels maintained between 8.00 and 9.50 mg/L. The fish were fed a commercial floating feed at approximately 1% of body weight twice daily at 08:00 and 16:00. Light/dark cycle of 14 h: 10 h. After acclimatization, feeding was stopped for two days, and the fish were randomly distributed into six culture tanks (2.0 m × 1.0 m × 1.0 m), with 100 fish per tank. The fish were randomly assigned into two groups: the treatment group and the control group, each with three replicates (three tanks per group). The treatment group was exposed to 10 ppt NaCl, a concentration determined from preliminary studies on the salt-alkali tolerance of *P. ussuriensis*. The control group was maintained in well-aerated tap water, under the same conditions as the acclimatization period, with no feeding during the experiment. The experimental period lasted 96 h, with sampling at time points of 6, 12, 24, 48, 72, and 96 h. For each time point, 9 fish from each replicate group were randomly selected, anesthetized with MS-22, and used for paraffin tissue sectioning and antioxidant enzyme activity analysis. At 24 h (In the salinity tolerance test, juveniles fish gradually exhibited stress responses within 30 min. Synchronous changes in gill physiological indicators were observed: gill filament hyperemia, increased amplitude of opercular movements, and a rapid rise in respiratory rate, indicating that the juveniles had activated an acute respiratory compensation mechanism. After 12 h of continuous salinity stress, the behavioral patterns of the juveniles underwent systematic changes—such as circular swimming along the tank walls, remaining suspended at the water surface, and an increased demand for dissolved oxygen—suggesting that the organisms had entered a compensatory adaptation stage involving energy metabolism and osmotic regulation. By 24 h, irreversible physiological failure was evident, characterized by tilting and rolling motions, increased mucus secretion on the body surface, startle reflexes triggered by external mechanical stimuli, and subsequent uncontrolled behaviors such as rapid swimming and rolling. Furthermore, a decrease in opercular movement frequency and a collapse of respiratory compensation, along with neuromuscular dysregulation, indicated that the organisms had exceeded their salinity tolerance threshold and entered a process of multisystem failure. Therefore, a 24-h time point was chosen for sampling.), 18 fish per group were randomly selected from each replicate for gill and kidney tissue sampling, which were rapidly frozen in liquid nitrogen and stored at −80 °C for subsequent RNA extraction, reverse transcription, sequencing, and metabolomics analysis. At the end of the experiment, 9 fish from each control group replicate were randomly selected for tissue sampling (for paraffin sectioning and antioxidant enzyme activity), and 18 fish were used for transcriptomics and metabolomics sequencing.

### Histopathological analysis and antioxidant enzyme activity assay

Gill and kidney tissues fixed in 4% paraformaldehyde were dehydrated in ethanol, cleared in xylene, embedded in paraffin, sectioned, stained with hematoxylin and eosin, and mounted. The tissue structures were then observed using an optical microscope.

Gill and kidney samples were homogenized in ice-cold physiological saline (1:9, w/v). The homogenates were then centrifuged at 2,500 g for 10 min at 4 °C, and the supernatant was collected to prepare a 10% tissue homogenate for measuring the activities of SOD, CAT, and glutathione peroxidase (GSH-Px), as well as MDA levels.Biochemical assays for catalase (CAT, A007-1-1), malondialdehyde (MDA, A003-1) superoxide dismutase (SOD, A001-3), and glutathione peroxidase (GSH-PX, A005-1) were performed according to the specifications provided by Nanjing Jian cheng Bioengineering Institute (Nanjing, China).The activities of GSH-Px, CAT, and SOD, as well as MDA levels, were measured using colorimetric methods at wavelengths of 405 nm, 405 nm, 450 nm, and 532 nm, respectively.

### Metabolomics analysis

A 20 mg sample was weighed into a 1.5 mL EP tube, to which two small steel beads were added. Then, 300 µL of methanol-water (V: V = 4:1, containing mixed internal standards, 4 µg/mL) was added. After pre-cooling at −40 °C for 2 min, the sample was ground using a grinder (60 Hz, 2 min). The sample was then extracted by sonication in an ice-water bath for 10 min and left to rest overnight at −40 °C. The sample was centrifuged at 12,000 rpm for 10 min at 4 °C, and 150 µL of the supernatant was collected. The supernatant was filtered using a 0.22 μm organic phase filter and transferred to an LC vial for analysis. Samples were stored at −80 °C until LC-MS analysis. Quality control (QC) samples were prepared by mixing equal volumes of the extraction solution from all samples. All extraction reagents were pre-cooled at −20 °C before use.

The analysis was performed using a Waters ACQUITY UPLC I-Class plus/Thermo QE ultra-performance liquid chromatography coupled with high-resolution tandem mass spectrometry (LC-MS) system.

### Transcriptome analysis

#### RNA extraction and library construction

Total RNA was extracted using TRIzol (Cat.No. B511311, Sangon Biotech, Shanghai, China) reagent according to the manufacturer’s instructions. RNA purity and concentration were assessed using a NanoDrop 2000 spectrophotometer (Thermo Scientific, USA), and RNA integrity was evaluated using an Agilent 2100 Bioanalyzer (Agilent Technologies, Santa Clara, CA, USA). Transcriptome libraries were constructed using the VAHTS Universal V6 RNA-seq Library Prep Kit following the manufacturer’s instructions. The library quality was verified using the Agilent 2100 Bioanalyzer, and sequencing was performed on the Illumina Novaseq 6000 platform to generate 150 bp paired-end reads. Transcriptome sequencing and analysis were conducted by Shanghai OE Biotech Co., Ltd. (Shanghai, China).

#### De novo transcriptome sequencing analysis and differential gene analysis

Raw reads in fastq format were processed using Trimmomatic to remove reads containing poly-N and low-quality reads, obtaining clean reads [26]. After removing adapters and low-quality sequences, clean reads were assembled into expressed sequence tags (contigs), and de novo transcript assembly was performed using Trinity [27]. The longest contig for each gene was selected as the unigene for further analysis.

Unigenes were annotated by alignment with databases including NCBI non-redundant (NR), Swiss-Prot, and Gene Ontology (GO), using Diamond software with an e-value threshold of < 1e-5 [28]. EggNOG and KOG annotations were also performed. Functional annotation of the unigenes was done based on sequence similarity to known proteins. These unigenes were mapped to the Kyoto Encyclopedia of Genes and Genomes (KEGG) database for pathway annotation, and GO classification was performed through mapping to Swiss-Prot and GO terms [29].

After unigene annotation, Bowtie2 was used to calculate the number of reads mapped to each unigene in each sample [30]. The expression levels of unigenes were calculated using eXpress [31] software and reported as fragments per kilobase of transcript per million mapped reads (FPKM). Differential expression was analyzed using DESeq2 [32], with negative binomial distribution (NB) for significance testing. The default criteria for differential expression were set at q-value < 0.05. Hierarchical clustering of differentially expressed genes (DEGs) was performed using R (v 3.2.0) to display gene expression patterns across different groups and samples. GO and KEGG pathway enrichment analysis of DEGs were performed using R, with visualization of enriched functional terms in bar charts or enrichment circle plots.

### Real-time quantitative PCR validation

Primers for differentially expressed genes were designed using Primer Premier 5 software, and synthesized by Sangon Biotech (Shanghai) Co., Ltd. (see table 1). β-actin was used as the internal reference gene. qRT-PCR was performed on 12 differentially expressed genes from the gill and kidney tissues. The primers and RT-qPCR conditions are detailed in Table 1. qRT-PCR was carried out using the TB Green^®^. Premix Ex Taq™ II (Tli RNaseH Plus) kit (Cat.No. RR820 A TaKaRa, Japan) following the manufacturer’s instructions on a real-time PCR system (Roche, Switzerland). Relative expression levels of DEGs were calculated using the 2^–ΔΔCt method [33].

**Table 1:** Sequences of primer pairs used in the qRT-PCR

Tissue	Gene name	Primer sequence(5’→3’)	Product length (bp)	Tm(℃)	Efffciency values(%)
gill	*MAFA*	F: GGACTGTGGCTGTAGGAGTTTGTG	106	67.8	98
		R: GGAACACCGACTGAAATCCAGACC		70.5	
	*MMP9*	F: TGGAAGAGACTGGTGAGCTGGAC	89	69.0	104
		R: TGTTTGGTAGTTGCGGATGTCTGG		70.7	
	*KCNQ1*	F: AGAGGGAGAGTGATGTGAGCAGTG	132	68.0	95
		R: AGCTGTCCTCAAGGGCAGATGG		70.7	
	*CXCL11*	F: GGACAACCAAGGACCAATGTGAGG	85	71.3	96
		R: CGATCTTGTCAATGCGTTGTGTCC		71.4	
	*KRT1*	F: GATTCAAACTGCCGTGACCGAAAC	103	71.2	103
		R: TTCCTCTTCCATTGCTTGCGTCTC		71.2	
	*LGMN*	F: AGCGTGTCGTTCTGGATCAATGC	83	71.3	93
		R: TGAGTTCGTCAGGCTTGCATGAAG		71.4	
kidney	*SOCS3*	F: TCCAGTCACCCACCACCTTCTTC	92	70.4	95
		R: CTGGCTTGGTTGTTGTGCTGTTG		70.7	
	*HMOX1*	F: GCAGCCCAAATCTCCTCGTAGC	127	69.9	105
		R: GAGAAGAATGCCACACCCTCCTTG		70.7	
	*ATP1 A*	F: CATGAAGCGGCAACCCAGAAATG	108	73.0	99
		R: AAGAACCCTGCTGCTGCTTGC		69.8	
	*HMGB1*	F: AGGACATTGCAGCATATCGGAAGG	110	70.6	97
		R: GTCGTCGTCGTCATCATCGTCATC		71.9	
	*IRF1*	F: AACCATGCCTGTGTCCAGAATGC	86	71.1	93
		R: CTACCCAGACAAGTCCAGCGATTG		69.6	
	*DDX58*	F: GCAGGCTCGGGTATGACTTTGAC	97	69.7	94
		R: CAGCAACGGAGGTGGCGATAAG		71.5	
*β*-actin		F: CCTCCGTCTGGATTTGGCTG	141	69.0	
		R: TCAAGGGCGACGTAGCAGAG		67.7	

### Integrated analysis of metabolomics and transcriptomics

Differential metabolites (DMs, *P*-value < 0.05,|VIP| > 1) and DEGs (q-value < 0.05, FC > 2.0 or FC < 0.5) were integrated for comparative analysis between control and stressed groups. Pearson correlation was calculated using the R package (stats package) to analyze the relationship between metabolomics and transcriptomics data. A heatmap was used to visualize the associations between DMs and DEGs.

### Data analysis

In this experiment, All data were expressed as mean ± SD. Statistical analysis was carried out with IBM SPSS Statistics 24.0, employing one-way analysis of variance (ANOVA) and the least significant difference (LSD) method to evaluate the significance of differences. Statistical significance was defined as *P* < 0.05 and is denoted by different lowercase letters. Graphs and charts were generated using the GraphPad Prism 8.3.0 software. All software and database lists used in this study are included in the Supplementary Material 1: Tables 1 and 2.

## Results

### Acute salinity stress on the tissue structure of *P. ussuriensis* juveniles

#### Changes in gill tissue structure

**Fig. 1:**
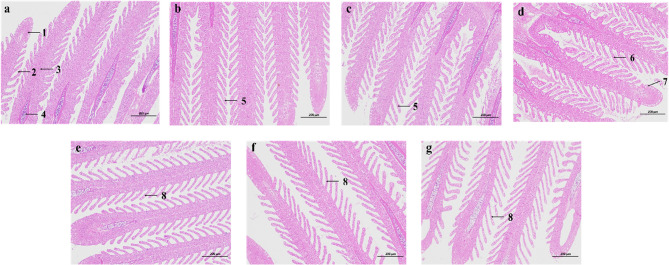
HE Staining Results of *P. ussuriensis* Gill Tissues Following Acute Salinity Stress Treatment at 6 h, 12 h, 24 h, 48 h, 72 h, and 96 h, and the Control Group. **a** Control group (0 h); **b** 6 h; **c** 12 h; **d** 24 h; **e** 48 h; **f** 72 h; **g** 96 h. Scale bar: 200 μm

The HE staining results are shown in Fig. 1. In the control group, the gill filaments, gill arches, and gill lamellae exhibited normal extension, with the tips of the gill filaments maintaining their typical morphology. At 6 and 12 h of salinity stress, no significant wrinkling was observed in the gill arches or filaments, though mild contraction of the gill lamellae was detected. After 24 h of acute salinity stress, while no notable wrinkling occurred in the gill arches or filaments, a distinct contraction and thinning of the gill lamellae became apparent. Additionally, the mucus cells along the margins of the gill filaments showed enlarged sizes. At 48, 72, and 96 h, both the gill filaments and lamellae displayed pronounced wrinkling and thinning.

#### Changes in kidney tissue structure

**Fig. 2:**
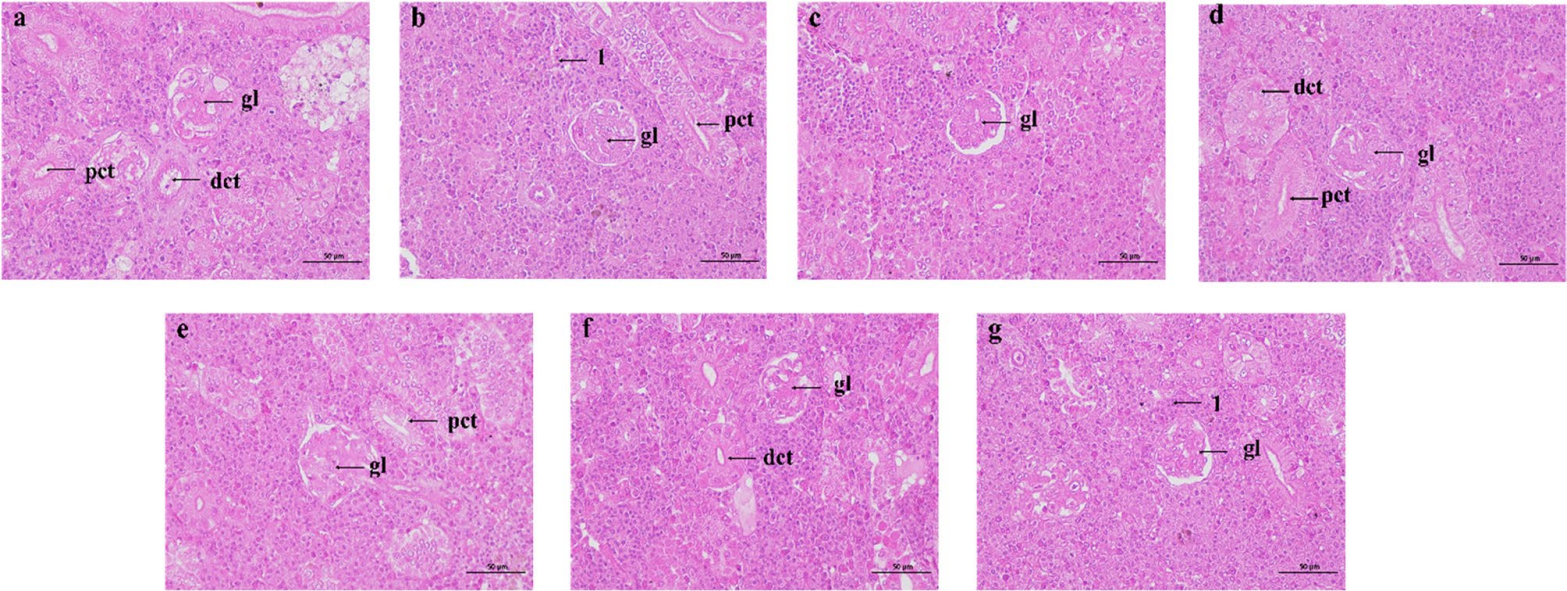
H&E Staining Results of *P. ussuriensis* Kidney Tissue Following Acute Salinity Stress Treatment at 6 h, 12 h, 24 h, 48 h, 72 h, and 96 h, and the Control Group. **a** Control group; **b** 6 h; **c** 12 h; **d** 24 h; **e** 48 h; **f** 72 h; **g** 96 h. PCT: Proximal convoluted tubule; DCT: Distal convoluted tubule; GL: Glomerulus; 1: Increased number of cells per field of view. Scale bar: 50 μm

As shown in the Fig. 2, in the control group, the glomeruli, proximal tubules, and distal tubules maintained normal cellular morphology. Under acute salinity stress, at 6, 12, 24, 48, 72, and 96 h, the glomeruli exhibited some degree of contraction, and the lumen appeared enlarged. Both the proximal and distal tubules showed a reduction in tubular diameter, with an increased number of cells observed within a single field of view.

### Changes in antioxidant enzyme activity of *P. ussuriensis* juveniles under acute salinity stress

**Fig. 3:**
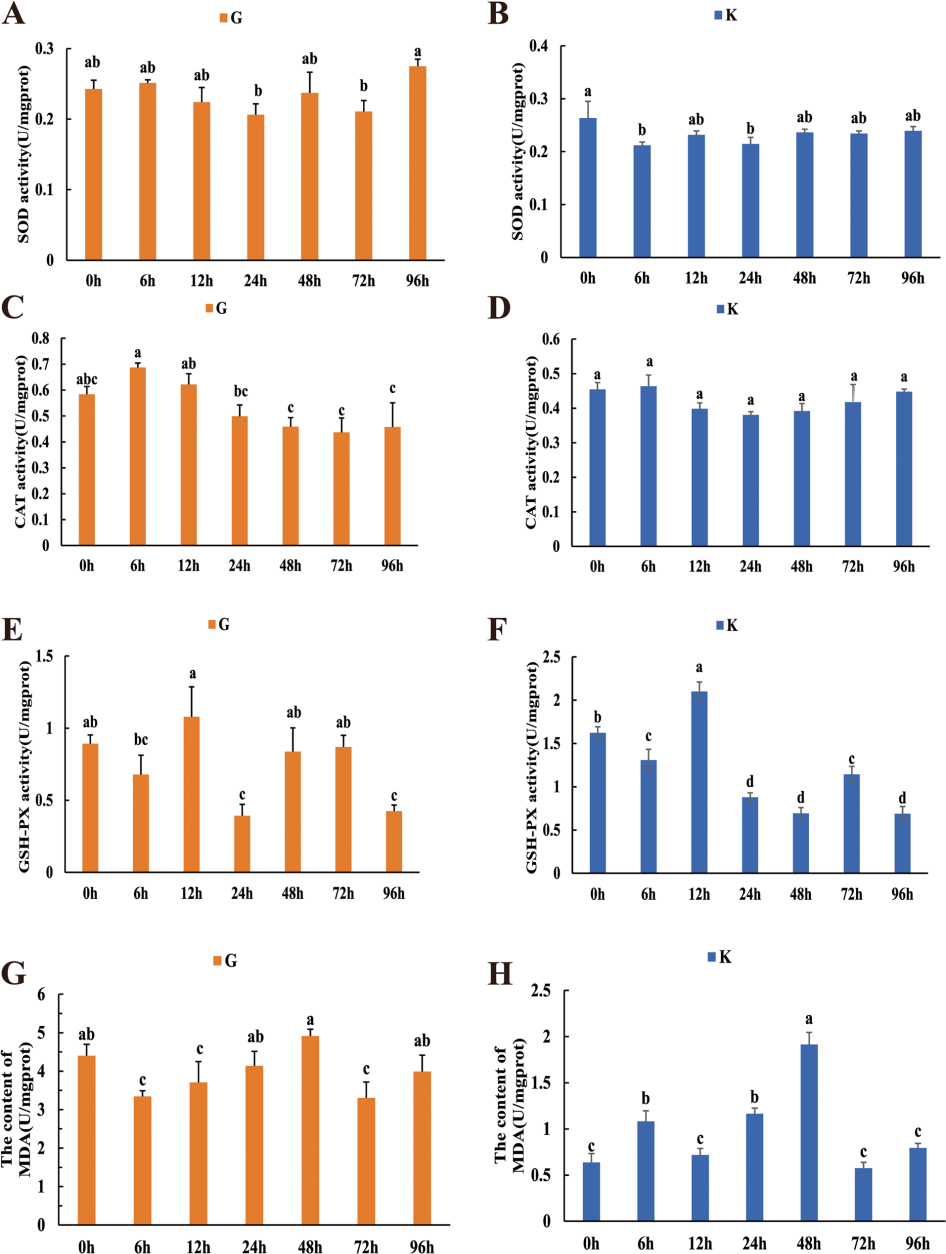
Changes in SOD, CAT, GSH-PX, MDA enzyme activities in gill (G) and kidney (K) of *P. ussuriensis* under acute salt stress at 0 h, 6 h, 12 h, 24 h, 48 h, 72 h, and 96 h. 0 h represents the control group. Different letters indicate significant differences (*P* < 0.05)

As shown in Fig. 3, the activities of antioxidant enzymes, including SOD, CAT, and GSH-Px, in the gill and kidney tissues of *P. ussuriensis* juveniles displayed temporal fluctuations in response to acute salinity stress. In both gill and kidney tissues, SOD activity initially decreased and then increased with the extension of the stress period. Specifically, kidney tissue showed significantly lower SOD activity at 6 h and 24 h compared to the control group. In gill tissue, SOD activity was significantly lower at 24 h and 72 h relative to the control group.

In both gill and kidney tissues, GSH-Px activity exhibited a fluctuating trend: it initially decreased, then increased, followed by another decline, and finally rose again under prolonged stress. Compared with the control group, GSH-Px activity in the gill tissues was significantly reduced at 6, 24, and 96 h. In the kidney tissues, GSH-Px activity was significantly higher than that in the control group at 12 h, but significantly decreased at the other time points. while gill tissue showed significantly higher enzyme activity at 48 h and 72 h compared to 24 h. MDA levels in gill tissue showed an initial decrease followed by an increase with longer stress exposure, while in kidney tissue, MDA levels initially increased and then decreased. MDA levels in gill tissue were much higher than in kidney tissue. Specifically, MDA levels in gill tissue were significantly lower than in the control group at 6 h, 12 h, and 72 h. In kidney tissue, MDA levels were significantly higher than the control group at 6 h, 24 h, and 48 h, with the highest MDA level observed at 48 h.

These results suggest that acute salinity stress leads to complex and time-dependent changes in antioxidant enzyme activities and oxidative stress markers in the gill and kidney tissues of *P. ussuriensis*. The differential response of these tissues indicates the varying capacities of the organs to cope with oxidative damage induced by salinity stress.

### Metabolomics analysis under salinity stress at 24 h

#### Multivariate statistical analysis of metabolites in gill and kidney tissues

**Fig. 4:**
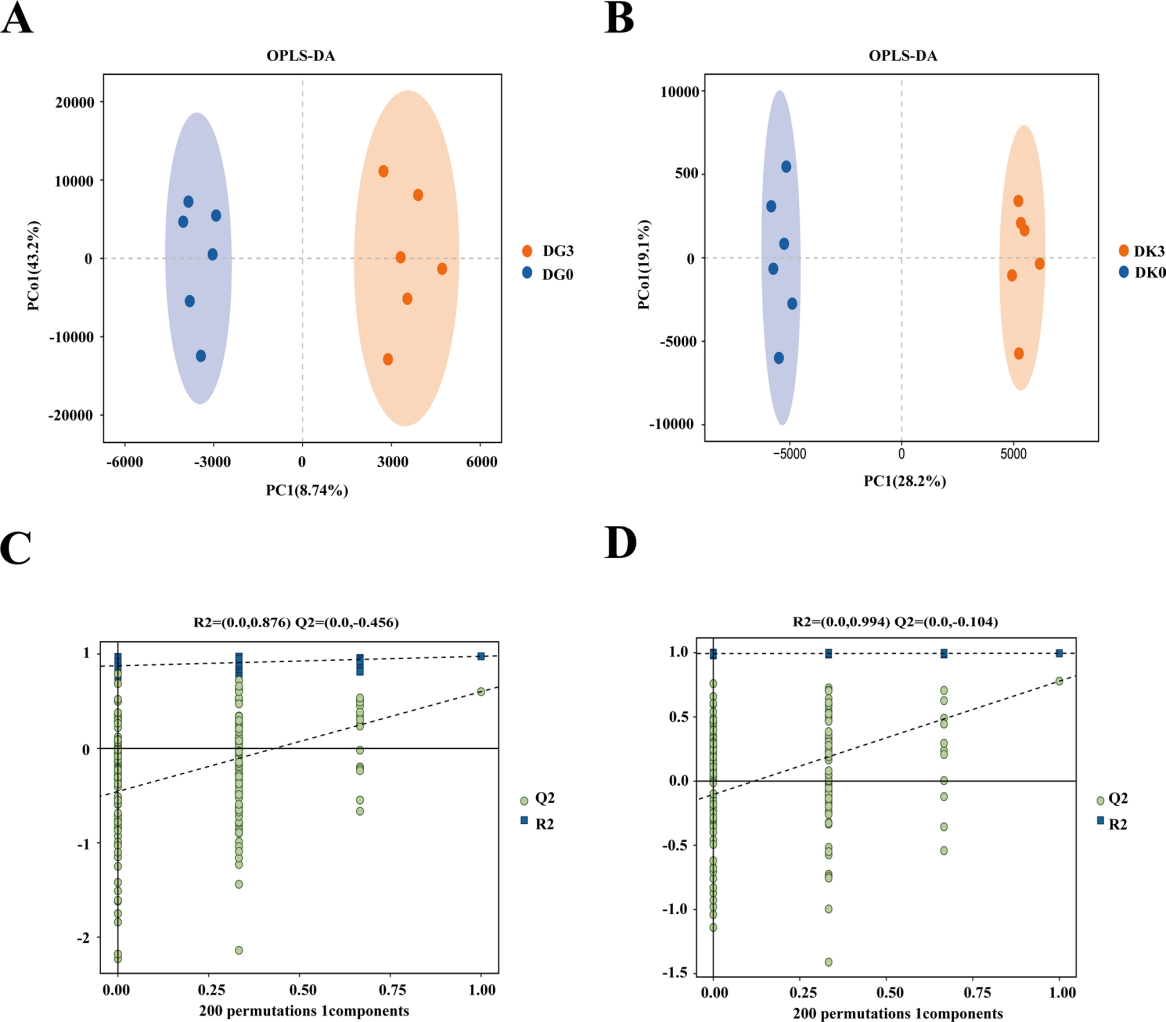
Quality analysis of metabolomics data. **A** OPLS-DA score plot of gill tissues. **B** OPLS-DA score plot of kidney tissues. **C** Permutation test plot of OPLS-DA for gill tissues. **D** Permutation test plot of OPLS-DA for kidney tissues

OPLS-DA (Orthogonal Partial Least Squares Discriminant Analysis) is a supervised model that differs from PCA (Principal Component Analysis) in that it reduces systematic noise and extracts variability-related information, making it a better model for classification. In the OPLS-DA score plots, the control and salinity-stressed groups were clearly separated, indicating that 24 h of salinity stress significantly disturbed the metabolite profiles in the gill tissue (Fig. 4A and B). The R^2^Y and Q^2^ values of the OPLS-DA model for gill tissue were 0.876 and − 0.456, respectively, while for kidney tissue, the values were 0.994 and − 0.104, demonstrating the high predictability and suitability of the OPLS-DA model for subsequent data analysis.

A permutation test with 200 iterations was performed on the OPLS-DA model to verify overfitting (Fig. 4C and D). The results showed that the R^2^ and Q^2^ values from the random permutations were higher than those from the original OPLS-DA model, confirming that the OPLS-DA model is reliable and effective, with no overfitting.

To identify significantly different metabolites between groups, a combination of multivariate and univariate analyses was employed. In OPLS-DA and PLS-DA analyses, the variable importance in projection (VIP) values were used to assess the impact and explanatory power of each metabolite in distinguishing between groups. Differential metabolites with biological significance were identified and validated using a t-test to confirm their significance.

**Fig. 5:**
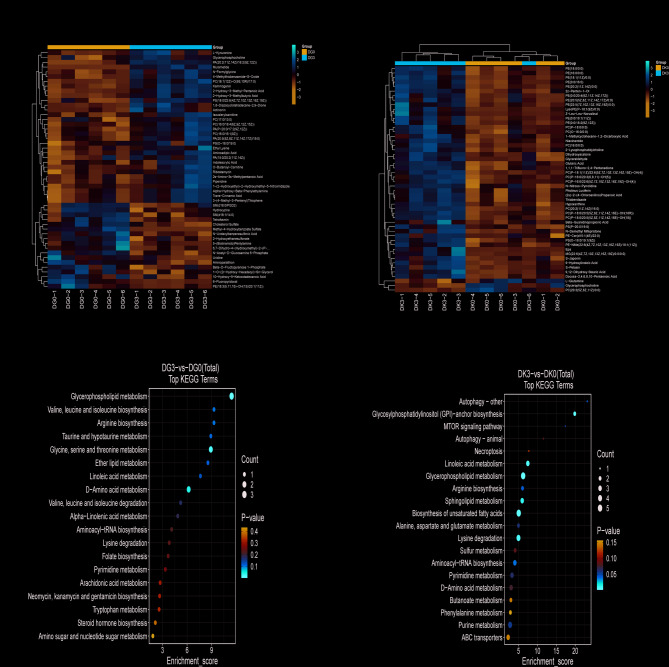
Hierarchical clustering analysis of DMs and metabolomics view of significant metabolic pathways. **A** Heatmap of top 50 metabolite clustering analysis for gill tissues. **B** Heatmap of top 50 metabolite clustering analysis for kidney tissues. **C** Total KEGG analysis bubble plot for gill tissues. **D** Total KEGG analysis bubble plot for kidney tissues. Relative metabolite levels are depicted according to the color scale, with red indicating up regulation and blue indicating down regulation. The size of the bubbles is proportional to the impact of each pathway, and the color of the bubbles indicates the degree of significance, ranging from highest (red) to lowest (white). DG0: Control group gill tissues; DG3: Experimental group gill tissues, 24 h salt stress; DK0: Control group kidney tissues; DK3: Experimental group kidney tissues, 24 h salt stress

With a VIP > 1.0 and a *p*-value < 0.05, a total of 85 differential metabolites (DMs) were identified in the gill tissue, with 49 upregulated and 36 downregulated, compared to the control group. In kidney tissue, 433 DMs were identified, with 252 upregulated and 181 downregulated. To visualize the relationships between the samples and the expression differences of metabolites across groups, Stratified cluster analysis was performed for the top 50 most significant metabolites based on all significant metabolite VIP scores (Fig. 5A and B). The results showed clear color changes between the groups, indicating that salinity stress significantly impacted the metabolite profiles of both gill and kidney tissues. Notably, lipid metabolism was most significantly affected.

To explore the potential metabolic pathways influenced by 24 h of salinity stress, pathway analysis was conducted using the KEGG database (website: https://www.genome.jp/kegg/), based on all differential metabolites (Total), upregulated differential metabolites (Up), and downregulated differential metabolites (Down). The results indicated that salinity stress for 24 h affected multiple metabolic pathways in both gill and kidney tissues. As shown in (Fig. 5C and D), the most significantly impacted metabolic pathways in gill tissue were glycerophospholipid metabolism, followed by glycine, serine, and threonine metabolism, D-amino acid metabolism, and the biosynthesis of valine, leucine, isoleucine, and arginine. In kidney tissue, the significantly affected pathway was glycosylphosphatidylinositol (GPI)-anchor biosynthesis, followed by linoleic acid metabolism, glycerophospholipid metabolism, and the biosynthesis of unsaturated fatty acids.

### Transcriptomic analysis of *P. ussuriensis* under 24-hour salinity stress

#### Overview of transcriptome sequencing results

A total of 12 samples were used for transcriptome sequencing in this analysis, generating 77.17 GB of clean data. The effective data for each sample ranged from 5.73 to 7.03 GB, with a Q30 base distribution between 94.84% and 95.46%, and an average GC content of 45.45%. A total of 84,887 unigenes were assembled, with a total length of 105,767,614 bp and an average length of 1,245.98 bp. The unigene annotation results were as follows: 38,479 (45.33%) unigenes annotated to the NR database, 29,227 (34.43%) annotated to the Swiss-Prot database, 14,046 (16.55%) annotated to the KEGG database, 22,444 (26.44%) annotated to the KOG database, 32,829 (38.67%) annotated to the eggNOG database, 26,268 (30.94%) annotated to the GO database, and 23,908 (28.16%) annotated to the Pfam database. The reads were aligned to the unigenes with a mapping rate ranging from 87.15 to 89.6%. Two comparison groups were established, and 2,554 and 1,066 DEGs were detected in the gill and kidney tissues, respectively. A total of 64,371 SSRs (simple sequence repeats) were predicted, with 14,770 unigenes containing more than one SSR. Additionally, 46,565 CDS sequences were predicted, of which 38,691 were predicted by the database alignment method, and 7,874 were predicted by ESTS can. The clean data set is available in the NCBI Sequence Read Archive (SRA), accession number to be provided.

#### DEG analysis

**Fig. 6:**
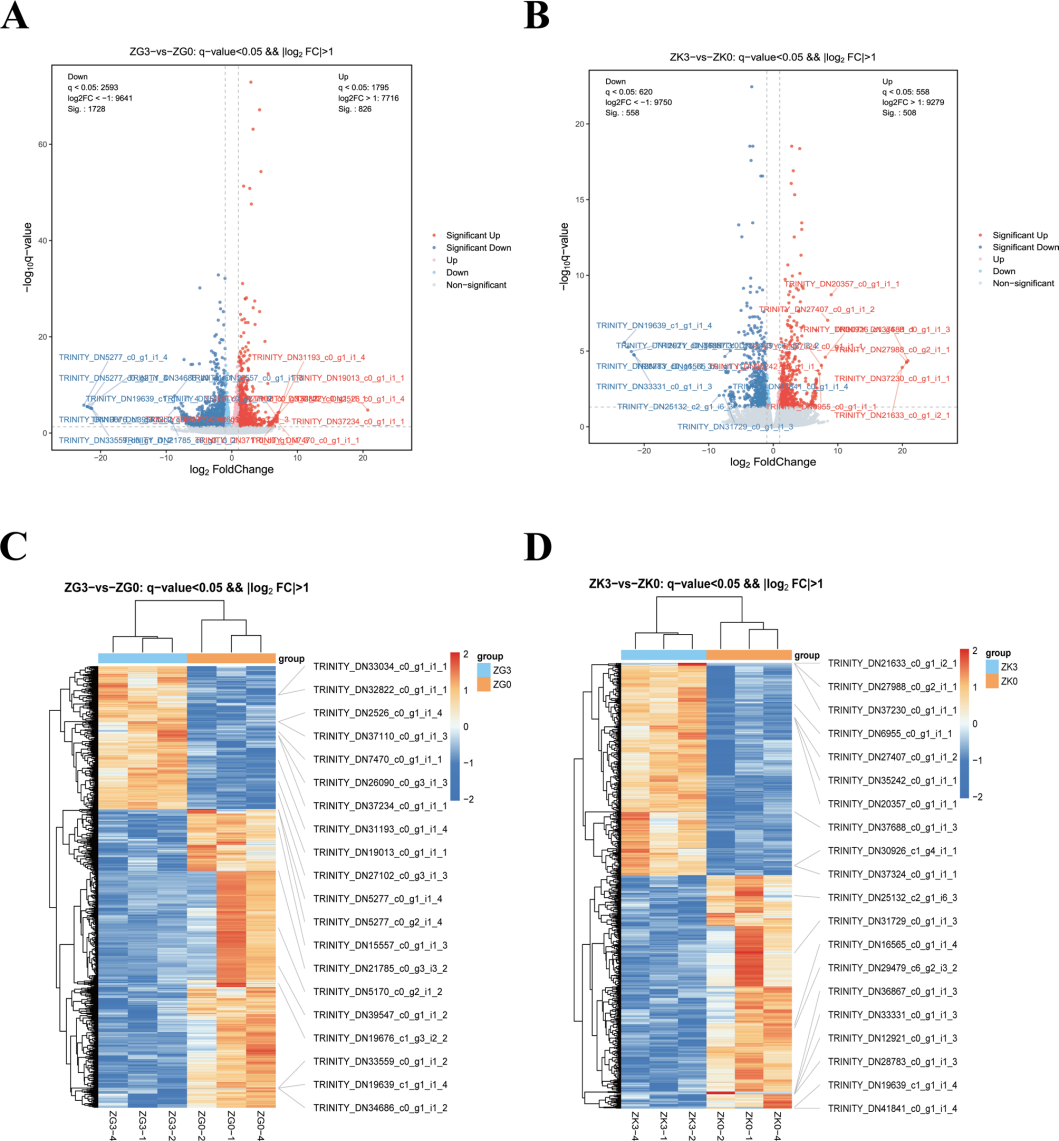
Volcano plots and clustering heatmaps of the distribution trends of DEGs in the stress and control groups of gill and kidney tissues. **A** and **B** Volcano plots showing the distribution trends of DEGs in gill and kidney tissues between the stress group and the control group. Red dots represent up regulated genes, blue dots represent down regulated genes, and gray dots represent genes with no differential expression. The X-axis displays log2 FoldChange, and the Y-axis displays -log10pValue. **C** and **D** Heatmap of clustering analysis for DEGs in gill and kidney tissues between the stress group and the control group. ZG0: Control group gill tissues; ZG3: Experimental group gill tissues, 24 h salt stress; ZK0: Control group kidney tissues; ZK3: Experimental group kidney tissues, 24 h salt stress

A total of 2,554 DEGs were identified in gill tissue and 1,066 in kidney tissue (with q < 0.05 and fold change > 2). Compared to the control group, 826 genes were upregulated, and 1,728 genes were downregulated in the gill tissue after 24 h of salinity stress. In kidney tissue, 508 genes were upregulated, and 558 genes were downregulated. Volcano plots were created to show the overall distribution of DEGs in both tissues (Fig. 6A and B). Hierarchical clustering analysis was performed on the DEGs, which revealed significant expression differences between the groups, with the results shown as heatmaps (Fig. 6C and D). The results indicate significant differences in gene expression between the control and salinity-stressed groups.

#### DEG pathway enrichment

**Fig. 7:**
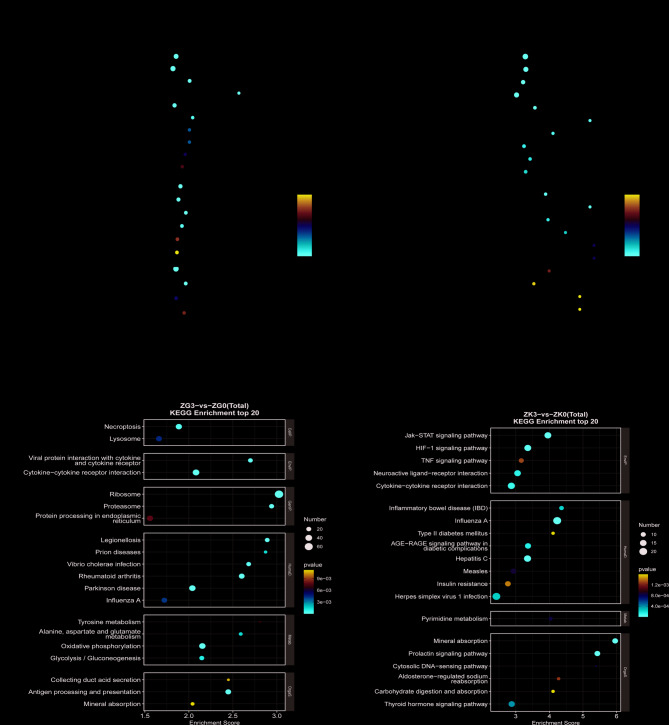
Bubble plots of GO and KEGG analysis of gill and kidney tissues. **A** and **B** GO analysis bubble plots of gill (G) and kidney (K) tissues. **C** and **D** KEGG analysis bubble plots of gill (G) and kidney (K) tissues

To better understand the biological significance of the DEGs, we used the GO database for comparison and annotation, as shown in (Fig. 7A and B). n the ZG3 vs. ZG0 group, biological process (BP) terms were significantly enriched in immune response, protein translation and folding, and leukocyte chemotaxis. Cellular component (CC) terms were primarily associated with the ribosome, cytosolic large ribosomal subunit, and proteasome complex. Molecular function (MF) analysis indicated significant enrichment in structural constituents of the ribosome and muscle. In contrast, in the ZK3 vs. ZK0 group, BP analysis highlighted immune-related functions such as defense response to viruses and innate immune response, as well as phosphate ion homeostasis. MF terms were predominantly associated with glucose transmembrane transporter activity, lipopolysaccharide binding, and other metabolic and immune regulatory functions.

Additionally, to further explore the relationship between DEGs and metabolic pathways, all DEGs were mapped to KEGG pathways, and the top 20 significant pathways were selected. KEGG enrichment analysis revealed that 24 h salinity stress significantly affected various metabolic and immune pathways in both gill and kidney tissues. In gill tissue, the significantly affected pathways included tyrosine metabolism, alanine, aspartate, and glutamate metabolism, oxidative phosphorylation, glycolysis, and gluconeogenesis. In kidney tissue, pyrimidine metabolism was the significantly affected pathway. Moreover, gill tissue was enriched in pathways related to cell necrosis, ribosome function, and antigen processing and presentation, while kidney tissue was enriched in pathways such as mineral absorption, aldosterone-regulated sodium reabsorption, prolactin and thyroid hormone signaling, Jak-STAT signaling, HIF-1 signaling, and TNF signaling pathways (Fig. 7C and D).

### qRT-PCR validation

To further validate the reliability of the transcriptomic analysis results, we performed qRT-PCR on 12 randomly selected DEGs from both the control and salinity stress groups. The specific genes selected for validation are listed in the Table 1. The results showed that the expression levels of the genes detected by qRT-PCR exhibited a similar trend to the RNA-Seq results (Supplementary Material 2). This consistency between qRT-PCR and RNA-Seq data confirms the reliability of our RNA-Seq findings. In gill tissue, the relative expression of *MAFA*,* MMP9*, and *KCNQ1* was upregulated, but the relative expression of *CXCL11*,* KRT1*, and *LGMN* showed a decreasing trend. In kidney tissue, the relative expression of *SOCS3* and *HMOX1* showed an increasing trend, while the relative expression of genes *ATP1 A*, *HMGB1*, *IRF1*, and *DDX58* was downregulated.

### Integrated analysis of metabolomics and transcriptomics

**Fig. 8:**
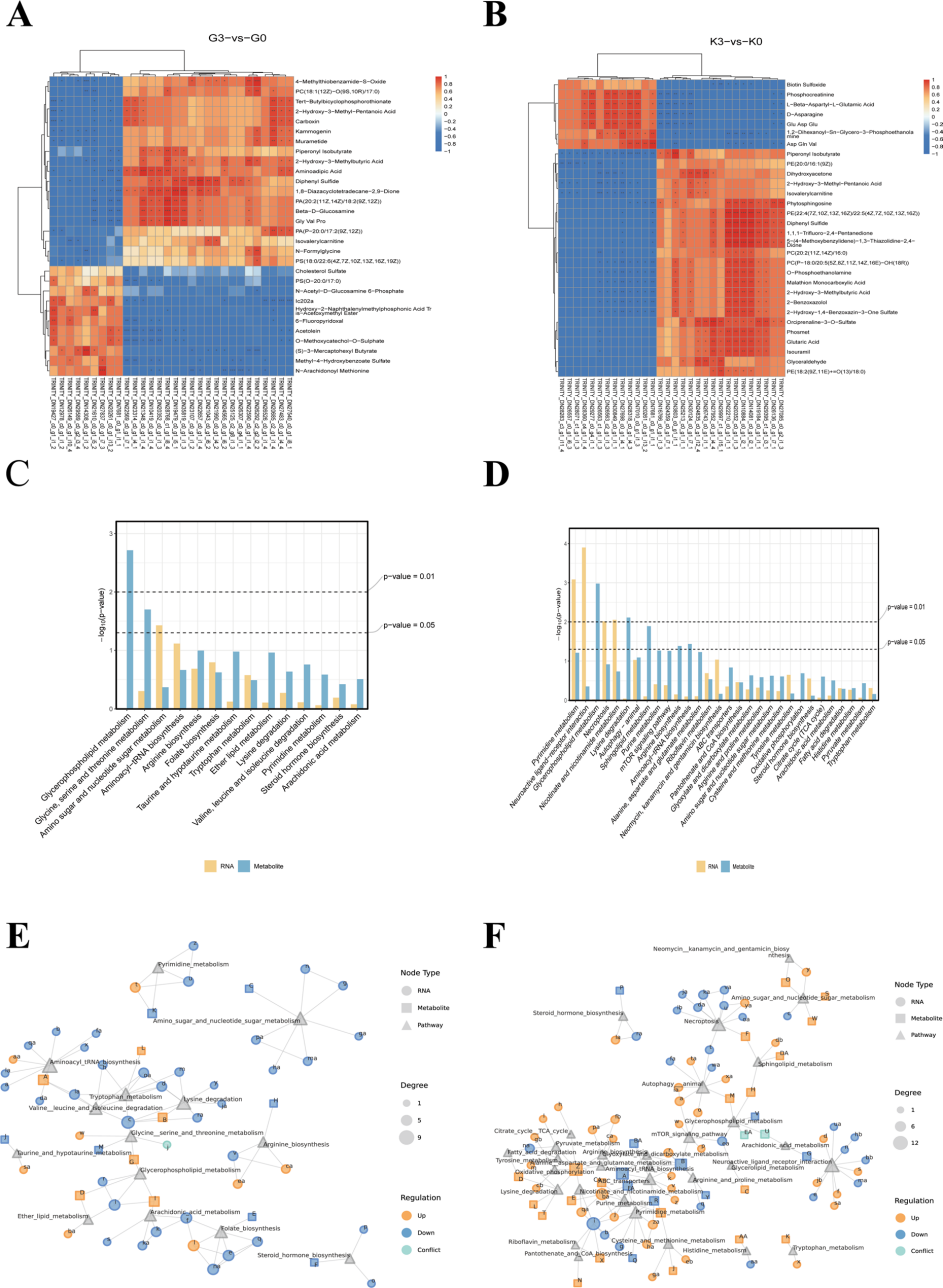
**A** and **B** Correlation heatmaps of the top differential metabolites in gill (G) and kidney (K) tissues. **C** and **D** KEGG pathway bar plots of gill (G) and kidney (K) tissues. **E** and **F** KGML network diagrams of gill (G) and kidney (K) tissues. Circular nodes represent differentially expressed genes/proteins, square nodes represent differential metabolites, and triangular nodes represent associated pathways. Node colors indicate regulatory patterns: orange for upregulated, blue for downregulated, and light blue for nodes with conflicting up- and down-regulation associations. Node size corresponds to the degree of connectivity

Correlation analysis using the Spearman correlation algorithm was conducted to explore the relationships between transcriptomics and metabolomics data. The top 30 significantly different entries (based on *p*-value ranking) from both the transcriptomic and metabolomic datasets were selected. For datasets with fewer than 30 significant entries, all significant results were used for correlation analysis. The correlation between these datasets was calculated, and the results are presented in heatmaps (Fig. 8A for gills and Fig. 8B for kidneys). The main contents are shown in the attached table pathwayG0-G3 (Supplementary Material 3) and pathwayK0-K3 (Supplementary Material 4).

Furthermore, a bar chart was created to display the common top 30 pathways from both omics, with significant threshold lines indicated (Fig. 8C and D). To further elucidate the network relationships between genes, metabolites, and pathways, a KGML (Kyoto Gene and Genomic Markup Language) network analysis was performed (Fig. 8E and F). The letters correspond to the relationships between metabolites and genes, as detailed in Supplementary Material 1: Table 3 − 1 and Table 3 − 2.

The integrated transcriptomic and metabolomic analysis revealed that metabolic activities in the gill tissue were significantly enriched in pathways related to glycerophospholipid metabolism, glycine, serine, and threonine metabolism, amino sugar and nucleotide sugar metabolism, aminoacyl-tRNA biosynthesis, arginine and folate biosynthesis, as well as the degradation of taurine, ether lipids, and branched-chain amino acids (valine, leucine, and isoleucine). Additionally, pathways such as pyrimidine metabolism, steroid hormone biosynthesis, and arachidonic acid metabolism were also involved.

In contrast, the kidney tissue exhibited a more extensive regulatory network. Besides sharing pathways with the gill, such as pyrimidine metabolism, glycerophospholipid metabolism, and steroid hormone biosynthesis, it was also enriched in neuroactive ligand-receptor interactions, programmed necrosis, autophagy, the mTOR signaling pathway, oxidative phosphorylation, the TCA cycle, and fatty acid degradation-key pathways associated with energy metabolism and cellular homeostasis. Furthermore, the kidney exhibited significant enrichment in the metabolism of multiple amino acids (e.g., alanine, aspartate, glutamate, cysteine, methionine, and tyrosine) and pathways related to transmembrane transport and cofactor synthesis, including ABC transporters and pantothenate/coenzyme A biosynthesis.

## Discussion

Salinity is a crucial factor in aquaculture, influencing fish stress responses, survival, and productivity. Research on acute salinity stress aids in optimizing salinity levels and breeding species suited for specific environments. This study integrates metabolomics, transcriptomics, and physiological-biochemical analyses to investigate the effects of acute salinity stress on tissue structure, energy metabolism, and immune pathways in *P. ussuriensis*, offering new insights into oxidative stress and regulatory mechanisms in the gill and kidney.

### Oxidative stress

SOD, CAT, GSH-Px, and MDA are key indicators of antioxidant stress, reflecting the cellular oxidative stress status and function of the antioxidant defense system [34]. MDA is a lipid peroxidation product generated via ROS-mediated degradation of membrane lipids, serves as a key biomarker for oxidative stress and membrane integrity disruption, reflecting cellular damage under pathological or environmental stress conditions [35]. After 24 h of salinity stress, kidney SOD and GSH-Px activities decreased significantly, while MDA levels increased. GSH-Px activity also declined in the gill, consistent with findings in *Cyprinus carpio* under similar conditions [36]. Similarly, in Oreochromis niloticus, MDA levels significantly increased after 5 and 10 days of 15 ppt salinity stress; whereas in Carassius auratus, SOD, CAT activity, and MDA levels significantly increased under 30-day alkalinity stress, and GSH-Px activity also increased significantly [21]. Differences in enzyme activities under oxidative stress may be linked to tissue function, antioxidant system activity, and metabolic stress. The gill, in direct contact with the environment, may have stronger antioxidant defenses, while the kidney, involved in excretion and detoxification, faces greater oxidative stress. Although CAT activity remained unchanged, this may be a temporary compensatory response, potentially increasing with intensified stress. *HMOX1* is a key oxidative stress-responsive gene [37]. It was significantly upregulated in this study, indicating a severe oxidative stress response under acute salinity stress, with insufficient antioxidant enzymes to fully counter the stress.

Oxidative stress also affects metabolism. Lipid peroxidation caused by oxidative stress not only damages cell membranes but also activates enzymes such as phospholipases, exacerbating inflammation and cellular damage [38]. Changes in glycerophospholipid metabolism also affect the antioxidant capacity of cells and their response to oxidative stress [39]. Metabolomics data showed that glycerophospholipid metabolism was most affected in the gill, while the GPI anchor biosynthesis pathway changed most in the kidney. Salinity-induced oxidative stress in *P. ussuriensis* leads to lipid peroxidation, membrane disruption, and oxidative damage to proteins and DNA, thereby impacting cell signaling and energy metabolism.

### Energy metabolism

Several studies have shown that salt and alkali exposure disturb lipid metabolism in aquatic organisms [21, 40]. For example, salinity stress in *Ictalurus punctatus* altered fatty acid, oxygen, and amino acid metabolism [41]. while in *Scophthalmus maximus*, pathways such as fat digestion and absorption, cholesterol metabolism, insulin resistance, and PPAR signaling were significantly affected under low-salinity exposure [42]. Additionally, low-salinity stress in *Seriola dumerili* led to marked changes in DEGs related to lipid and vitamin metabolism, ion transport, and signal transduction [43]. In this study, 24 h of salinity stress significantly impacted multiple metabolic pathways in both gill and kidney tissues. In the gill, the most significantly altered pathway was glycerophospholipid metabolism, followed by glycine, serine, and threonine metabolism; d-amino acid metabolism; and the biosynthesis of valine, leucine, isoleucine, and arginine. In the kidney, GPI anchor biosynthesis was the most affected pathway, followed by linoleic acid metabolism, glycerophospholipid metabolism, and the biosynthesis of unsaturated fatty acids. Through the integrated analysis of metabolome and transcriptome, we found that salinity changes may regulate metabolic changes by regulating PC, PA, PE, and SM, as well as changes in some key signaling pathways.

Metabolomic analysis provided a comprehensive profile of metabolic changes in the gill and kidney tissues of *P. ussuriensis* juveniles under acute salinity stress. Compared to the control, the salinity stress group exhibited significantly increased levels of phosphatidylcholine (PC) and phosphatidylethanolamine (PE) in the kidney, and elevated phosphatidic acid (PA) and PC in the gill. In contrast, sphingomyelin (SM) levels were markedly reduced. Notably, PC in the kidney showed two distinct trends, indicating differential metabolic responses. As the major components of cellular membranes, PC, PA, PE, and SM play crucial roles in activating signaling pathways, regulating enzyme activity, and maintaining lipid transport and metabolic balance [44]. Phosphatidic acid (PA) serves as a precursor for several phospholipids, such as PC and phosphatidylinositol, and is involved in fatty acid synthesis and storage. Phosphatidylethanolamine (PE) is a key precursor for multiple phospholipids, including PC, thereby influencing the dynamic balance of intracellular lipids and facilitating membrane fusion with internal structures [45]. Sphingomyelin (SM), predominantly located in nerve cell membranes and myelin, is tightly regulated by its metabolic enzymes. Specifically, sphingomyelinase (SMase) hydrolyzes SM into ceramide (Cer), a bioactive molecule that modulates cell proliferation, growth, and apoptosis, while SM synthase (SMS) and related enzymes maintain the balance between SM and Cer to ensure cellular homeostasis [46].

Transcriptomic analysis supported these findings, with gill tissue enriched in pathways including tyrosine metabolism; alanine, aspartate, and glutamate metabolism; oxidative phosphorylation; glycolysis; and gluconeogenesis, and kidney tissue showing enrichment in pyrimidine metabolism pathways. In addition, the insulin signaling pathway—critical for blood glucose regulation and energy balance [47]. was significantly enriched in both gill and kidney tissues. In the gill, the HIF-1 signaling pathway and insulin-regulating transcription factor *MAFA* were upregulated, while *PPP1R3*, a key regulator of glucose and lipid metabolism, was downregulated. In the kidney, *SLC34 A*, which influences ATP synthesis and cellular energy status through phosphate regulation [48], was downregulated, whereas *EGLN*, involved in hypoxia-inducible factor degradation [49], was upregulated. These findings indicate that acute salinity stress markedly disrupts energy metabolism and blood glucose regulation in *P. ussuriensis*.

These findings suggest that acute salinity stress triggers lipid metabolic dysregulation and adaptive membrane remodeling, with gill tissues exhibiting heightened vulnerability compared to kidneys. This disparity likely stems from the gill’s direct environmental exposure and dual roles in osmoregulation/detoxification, rendering it prone to oxidative and metabolic injury under abrupt salinity shifts.

### Immune response

Prior studies indicate salinity stress differentially impacts immune responses in fish gills and kidneys under acute exposure [21]. Here, transcriptomic analysis revealed gill-specific enrichment of pathways linked to cell necrosis, ribosomal activity, and antigen processing/presentation. Notably, *HMGB1* is a nuclear DAMP (danger-associated molecular pattern) activating inflammation via TLR/NF-κB pathways—was downregulated, potentially attenuating inflammatory responses by suppressing TLR/RAGE-NF-κB signaling. Concurrently, *MMP9* (extracellular matrix remodeling [50]. *CXCL11* (immune cell recruitment [51]) and *LGMN* (antigen processing [52]) were upregulated, collectively driving tissue homeostasis remodeling while dampening immune activation. These shifts were associated with TGF-β/Smad/NF-κB pathway enrichment in gills under 24 h 10 ppt salinity, suggesting a coordinated mechanism balancing tissue repair and inflammation resolution.

In kidney tissues, enrichment was found in the JAK-STAT and TNF signaling pathways. The JAK-STAT pathway, activated by cytokines such as interferons and interleukins, regulates immune cell differentiation and the balance between pro- and anti-inflammatory factors [53]. TNF-α signaling, mediated via NF-κB, triggers inflammation and cell survival, and influences cell proliferation and apoptosis through p38, ERK, and JNK pathways [54]. *SOCS3*, a cytokine signaling inhibitor, suppresses JAK-STAT signaling by binding to JAK kinases and preventing STAT translocation, while *IRF1* regulates the expression of immune-related genes, including IFN-stimulated genes [55, 56]. Transcriptomic data from this study revealed that under 24 h of 10 ppt salinity stress, kidney tissues exhibited activation of the JAK-STAT and TNF pathways, with *SOCS3* upregulated and IRF1 downregulated. These changes likely help to prevent excessive immune activation and apoptosis while maintaining controlled inflammation.

Transcriptomic data revealed that 24 h salinity stress triggered inflammatory immune responses in both gill and kidney tissues of *P. ussuriensis*, transitioning from acute to chronic inflammation, with kidneys exhibiting delayed activation compared to gills. This aligns with prior findings: in *Carassius auratus*, saline-alkali stress activated IL-17/NF-κB signaling in gills [21], while high salinity suppressed inflammation in *Seriola dumerili*, impairing pathogen clearance [43]. Integrated omics analyses identified downregulation of key detoxification/energy metabolism genes in gills. *ALDH* and *ALDH7 A1* are primarily involved in aldehyde metabolism and oxidative stress response; *AOX* plays a key role in the oxidation of aldehydes and heterocyclic compounds, important for the metabolism of drugs and endogenous molecules [57]; *HADHA* is crucial for the β-oxidation of long-chain fatty acids, mainly involved in energy production in the mitochondria [58]. *ALDH* enzymes help detoxify reactive aldehydes during oxidative stress [59]. However, chronic oxidative stress may downregulate the expression of *ALDH* and *ALDH7 A1*, thereby reducing metabolism and exacerbating chronic inflammation. I Chronic oxidative stress may suppress *ALDH/ALDH7 A1* expression, exacerbating inflammation by accumulating reactive aldehydes. Pro-inflammatory cytokines (e.g., *TNF-α*, *IL-6*) could further inhibit *ALDH* activity to sustain immune signaling via aldehyde-mediated DAMP pathways [60]. Collectively, these dysregulations impair detoxification, lipid catabolism, and energy homeostasis in gills, highlighting tissue-specific vulnerability under salinity stress.

In kidney tissue, *ENPP1/3*, lglutamine (downregulated), and succinic acid (upregulated) showed notable changes. *ENPP1* and *ENPP3*, part of the ectonucleotide pyrophosphatase/phosphodiesterase family, regulate extracellular adenosine levels and inflammation, linking intercellular signaling with immune regulation [61]. Glutamine plays a key role in carbon skeleton synthesis, amino acid metabolism, nitrogen balance, the TCA cycle, and antioxidant production (e.g., glutathione), and is especially important for rapidly proliferating cells [62]. Succinic acid, a central TCA cycle intermediate, is produced during aerobic metabolism and modulates inflammatory responses by inhibiting protein decarboxylase activity [63]. The findings suggest that under acute salinity stress, kidney cells experience reduced antioxidant defense and metabolic adaptability, potentially entering a state of inflammation or oxidative stress with a shift toward anaerobic or inflammatory metabolic pathways.

## Conclusion

This study employed LC-MS/MS metabolomics, transcriptomics, and physiological-biochemical assays to delineate the molecular and systemic impacts of 24 h acute salinity stress (10 ppt NaCl) on *P. ussuriensis* juveniles. Both gill and kidney tissues exhibited severe oxidative damage, lipid-energy metabolic dysregulation, and immune-inflammatory activation, with gills showing earlier and more pronounced responses due to direct environmental exposure. Notably, tissue-specific adaptive strategies emerged: gills prioritized membrane lipid remodeling and acute-phase inflammation resolution (e.g., TGF-β/Smad/NF-κB pathway modulation), while kidneys delayed inflammatory transitions through autophagy-apoptosis balancing and TCA cycle reprogramming. Multi-omics integration revealed a “stress-to-chronicity” shift characterized by sustained downregulation of detoxification enzymes (*ALDH7 A1*, *AOX*, *HADHA*) and pro-inflammatory mediator accumulation (*HMGB1*, *MMP9*), exacerbating metabolic-immune crosstalk. These findings uncover a dynamic tolerance mechanism balancing osmotic adaptation and redox homeostasis, providing actionable targets for salt-resistant breeding programs. The study underscores the necessity of combining omics-driven molecular insights with physiological validation to decode aquatic stress adaptation comprehensively.

## Supplementary Information

Supplementary Material 1.

Supplementary Material 2.

Supplementary Material 3.

Supplementary Material 4.

## Data Availability

The raw sequencing reads from the RNA-seq described in this study have been deposited in the NCBI Sequence Read Archive under accession number [PRJNA1242274] (https://www.ncbi.nlm.nih.gov/bioproject/PRJNA1242274).
