# Supplementary material for: Comprehensive multi-omics and biochemical analysis to elucidate the molecular response mechanisms of gill and kidney tissues under acute salinity stress in Pseudobagras ussuriensis
Source: BMC Genomics. 2025 Jul 1;26:590. doi: 10.1186/s12864-025-11773-w (PMC12211720; doi:10.1186/s12864-025-11773-w)
Supplement: Supplementary file 1 — Supplementary Material 1. [file 12864_2025_11773_MOESM1_ESM.docx]

**Supplementary Material 1**

**Table 1: Databases and links used in de novo transcriptome analysis**

| Databases | links |
| --- | --- |
| eggNOG Database | http://eggnog6.embl.de/ |
| GO Database | http://geneontology.org/ |
| KEGG Database | http://www.genome.jp/kegg/ |
| KOG Database | ftp://ftp.ncbi.nih.gov/pub/COG/KOG |
| NR Database | ftp://ftp.ncbi.nlm.nih.gov/blast/db/FASTA/nr.gz |
| Pfam Database | https://www.ebi.ac.uk/interpro/ |
| Swissprot Database | https://www.uniprot.org/ |

**Table 2: Software tools and their functions in this study, From Network Analysis to Data Visualization.**

| Software | Version | Parameters | Function |
| --- | --- | --- | --- |
| fastp | 0.20.1 | --length_required 50 | Raw reads quality control |
| Trinity | 2.4.0 | --seqType fq --SS_lib_type RF | Sequence assembly |
| CD-HIT | 4.6 | default | Redundant sequence removal |
| bowtie2 | 2.3.3.1 | --reorder -k30 -t | Transcript alignment |
| eXpress | 1.5.1 | --rf-stranded | Quantification |
| DESeq2 | 1.20.0 | qvalue<0.05, \|log2FoldChange\|>1 | Differential analysis |
| DESeq | 1.18.0 | pvalue<0.05, \|log2FoldChange\|>1 | Differential analysis |
| MISA | - | - | SSR identification |
| Primmer3 | - | - | SSR analysis |
| ESTScan | 3.0.3 | default | CDS prediction |
| python | 3.9.17 |  | Analysis framework |
| R | 4.3.2 |  | Visualization |
| ggplot2 | 3.4.3 |  | Visualization |
| matplotlib | 3.8.0 |  | Heatmap, density plot |
| NetworkX | 3.1 |  | Network diagram |

**Table 3-1** **Table of Pathway-Metabolite-Gene Relationships in Gill Tissue**

| Metabolic Pathway | Letter Code | KEGG ID Metabolite | regulation |
| --- | --- | --- | --- |
| Pyrimidine_metabolism | z | ndk, NME | down |
|  | u | CMPK2 | down |
|  | t | E3.1.3.5 | up |
|  | K | Uridine | down |
| Amino_sugar_and_nucleotide_sugar_metabolism | n | glmS, GFPT | down |
|  | g | GPI, pgi | down |
|  | ga | UGP2, galU, galF | down |
|  | ma | UAP1 | down |
|  | ha | TSTA3, fcl | down |
|  | pa | CHS1 | down |
|  | C | N-Acetyl-D-Glucosamine 6-Phosphate | down |
| Arginine_biosynthesis | ca | glsA, GLS | up |
|  | ea | glnA, GLUL | up |
|  | v | GLUD1_2, gdhA | down |
|  | H | N-Acetyl-L-Glutamic Acid | down |
| Steroid_hormone_biosynthesis | p | COMT | down |
|  | o | HSD11B2 | down |
|  | F | Cholesterol Sulfate | down |
| Folate_biosynthesis | q | PCBD, phhB | down |
|  | e | QDPR | down |
|  | na | phhA, PAH | down |
|  | l | DHFR, folA | up |
|  | f | CBR1 | down |
|  | E | Sepiapterin | down |
| Arachidonic_acid_metabolism | f | CBR1 | down |
|  | k | PTGES3 | down |
|  | ka | HPGDS | down |
|  | s | GPX4 | down |
|  | I | PC(16:0/18:4(6Z,9Z,12Z,15Z))\nPC(16:0/16:1(9Z)) | up |
| Glycerophospholipid_metabolism | I | PC(16:0/18:4(6Z,9Z,12Z,15Z))\nPC(16:0/16:1(9Z)) | up |
|  | D | Glycerophosphocholine | up |
|  | G | PS(18:0/22:6(4Z,7Z,10Z,13Z,16Z,19Z)) | up |
|  | i | PLD3_4 | down |
|  | r | LPIN | down |
| Ether_lipid_metabolism | ba | ENPP2 | up |
|  | i | PLD3_4 | down |
|  |  |  |  |
|  | D | Glycerophosphocholine | up |
| Glycine__serine_and_threonine_metabolism | j | PGAM, gpmA | conflict |
|  | M |  | down |
|  | w | AOC3, AOC2, tynA | up |
|  | c | ALDH7A1 | down |
| Lysine_degradation | ra | AASS | down |
|  | ja | EHMT | down |
|  | y | SMYD | down |
|  | m | DLST, sucB | down |
|  | d | HADHA | down |
|  | oa | ALDH | down |
|  | B | Aminoadipic Acid | up |
| Tryptophan_metabolism | d | HADHA | down |
|  | m | DLST, sucB | down |
|  | oa | ALDH | down |
|  | h | AOX | down |
|  | c | ALDH7A1 | down |
|  | ia | IL4I1 | down |
| Valine__leucine_and_isoleucine_degradation | c | ALDH7A1 | down |
|  | ia | IL4I1 | down |
|  | h | AOX | down |
|  | oa | ALDH | down |
|  | d | HADHA | down |
|  | A | 2s-Amino-3s-Methylpentanoic Acid | up |
| Aminoacyl_tRNA_biosynthesis | x | SARS, serS | down |
|  | fa | KARS, lysS | down |
|  | b | QARS, glnS | down |
|  | qa | RARS, argS | down |
|  | aa | DARS | up |
|  | la | TARS, thrS | down |
|  | a | HARS, hisS | down |
|  | da | YARS, tyrS | down |
| Taurine_and_hypotaurine_metabolism | sa | CDO1 | up |
|  | J | 2-Hydroxyethanesulfonate | down |

**Table 3-2** **Table of Pathway-Metabolite-Gene Relationships in Kidney Tissue**

| Metabolic Pathway | Letter Code | KEGG ID Metabolite | regulation |
| --- | --- | --- | --- |
| Steroid_hormone_biosynthesis | P | Estrone 3-Glucuronide | down |
|  | ra | SULT2B1 | down |
|  | la | CYP7A1 | up |
| Necroptosis | ib | PYG, glgP | down |
|  | ja | CAPN2 | down |
|  | da | STAT3 | down |
|  | ka | IRF9, ISGF3G, P48 | down |
|  | va | STAT1 | down |
|  | u | CASP1 | down |
|  | ya | IL1B | up |
|  | oa | NOX2, GP91, CYBB | down |
|  | t | HMGB1 | down |
|  | F | SM(d18:1/24:1(15Z)) | up |
| Neomycin__kanamycin_and_gentamicin_biosynthesis | y | HK | up |
|  | O | Udp-Glcnac | up |
| Amino_sugar_and_nucleotide_sugar_metabolism | S | Udp-N-Acetyl-D-Mannosamine | up |
|  | W | N-Acetylneuraminic Acid 9-Phosphate | up |
|  | c | E3.2.1.14 | down |
| Sphingolipid_metabolism | F | SM(d18:1/24:1(15Z)) | up |
|  | db | CERS1_2_3_4, LASS1_2_3_4 | up |
|  | DA | Phytosphingosine | up |
|  | H | O-Phosphoethanolamine | up |
| Autophagy___animal | fa | RAB1A | down |
|  | ta | RAB33B | up |
|  | wa | ERN1 | down |
|  | xa | CTSL | up |
|  | o | DDIT4, REDD1 | up |
|  | a | PIK3R1_2_3 | up |
|  | ia | IRS2 | up |
|  | M | PE(22:4(7Z,10Z,13Z,16Z)/22:5(4Z,7Z,10Z,13Z,16Z))\nPE(20:0/16:1(9Z)) | up |
| Glycerophospholipid_metabolism | ea | LPIN | down |
|  | M | PE(22:4(7Z,10Z,13Z,16Z)/22:5(4Z,7Z,10Z,13Z,16Z))\nPE(20:0/16:1(9Z)) | up |
|  | H | O-Phosphoethanolamine | up |
|  | V | Glycerophosphocholine | down |
|  | U | PC(20:2(11Z,14Z)/16:0)\nPC(20:2(11Z,14Z)/P-18:1(11Z))\nPC(15:0/18:1(11Z))\nPC(15:0/15:0)\nPC(15:0/18:4(6Z,9Z,12Z,15Z)) | conflict |
|  | EA | PC(16:0/0:0)\nPC(17:0/0:0)\nPC(P-18:1(9Z)/0:0)\nPC(P-18:0/0:0)\nPC(20:3(5Z,8Z,11Z)/0:0) | conflict |
|  | ea | LPIN | down |
| Arachidonic_acid_metabolism | U | PC(20:2(11Z,14Z)/16:0)\nPC(20:2(11Z,14Z)/P-18:1(11Z))\nPC(15:0/18:1(11Z))\nPC(15:0/15:0)\nPC(15:0/18:4(6Z,9Z,12Z,15Z)) | conflict |
|  | G | Pgd2-D4 | down |
| Neuroactive_ligand_receptor_interaction | G | Pgd2-D4 | down |
|  | d | PTH | up |
|  | ua | P2RX5 | down |
|  | hb | CGA | down |
|  | n | PTGER4 | down |
|  | bb | LTB4R1 | down |
|  | s | AGT, SERPINA8 | up |
|  | ma | PTGER1 | down |
|  | f | GLP2R | up |
|  | sa | TBXA2R | up |
|  | jb | P2RY4 | up |
|  | e | ADM | down |
| Glycerolipid_metabolism | C | Dihydroxyacetone | up |
|  | ea | LPIN | down |
| mTOR_signaling_pathway | w | SGK1 | up |
|  | a | PIK3R1_2_3 | up |
|  | ia | IRS2 | up |
|  | o | DDIT4, REDD1 | up |
|  | p | PRKCA | up |
|  | B | L-Arginine | down |
| Arginine_and_proline_metabolism | Y | N2-Succinyl-L-Ornithine | down |
|  | B | L-Arginine | down |
|  | r | E3.5.3.1, rocF, arg | up |
|  | q | E2.7.3.2 | down |
| Tryptophan_metabolism | x | TDO2, kynA | up |
|  | K | 5-Hydroxyindoleacetic Acid | up |
| Histidine_metabolism | aa | HNMT | down |
|  | AA | Imidazolepropionic Acid | up |
| Cysteine_and_methionine_metabolism | I | Sulfate | up |
|  | ga | CBS | up |
|  | j | CECR1, ADA2 | up |
|  | eb | metK | up |
| Pantothenate_and_CoA_biosynthesis | N | Pantetheine | up |
|  | i | ENPP1_3, CD203 | down |
| Riboflavin_metabolism | i | ENPP1_3, CD203 | down |
|  | X | Fad | up |
| Pyrimidine_metabolism | Q | Pseudouridine | down |
|  | R | Uridine | down |
|  | A | L-Glutamine | down |
|  | ha | deoA, TYMP | up |
|  | za | udp, UPP | up |
|  | b | CMPK2 | down |
|  | g | CMPK1, UMPK | down |
|  | i | ENPP1_3, CD203 | down |
| Purine_metabolism | A | L-Glutamine | down |
|  | FA | Guanosine | down |
|  | CA | Hypoxanthine | up |
|  | I | Sulfate | up |
|  | j | CECR1, ADA2 | up |
|  | i | ENPP1_3, CD203 | down |
|  | ba | punA, PNP | up |
| Nicotinate_and_nicotinamide_metabolism | E | Niacinamide | up |
|  | Z | Succinic Acid | up |
|  | qa | NRK1_2 | up |
|  | i | ENPP1_3, CD203 | down |
|  | ba | punA, PNP | up |
| ABC_transporters | BA | L-Phenylalanine | down |
|  | B | L-Arginine | down |
|  | I | Sulfate | up |
|  | R | Uridine | down |
|  | FA | Guanosine | down |
|  | A | L-Glutamine | down |
|  | z | ABCC12 | up |
|  | v | ABCC9, SUR2 | up |
|  | ba | punA, PNP | up |
| Aminoacyl_tRNA_biosynthesis | A | L-Glutamine | down |
|  | B | L-Arginine | down |
|  | BA | L-Phenylalanine | down |
|  | k | DARS | up |
| Glyoxylate_and_dicarboxylate_metabolism | r | E3.5.3.1, rocF, arg | up |
|  | B | L-Arginine | down |
|  | A | L-Glutamine | down |
| Alanine__aspartate_and_glutamate_metabolism | Z | Succinic Acid | up |
|  | A | L-Glutamine | down |
|  | ca | ASRGL1, iaaA | up |
| Arginine_biosynthesis | Z | Succinic Acid | up |
|  | A | L-Glutamine | down |
|  | pa | glyA, SHMT | up |
|  | fb | GLDC, gcvP | up |
| Lysine_degradation | m | ASH1L | up |
|  | Z | Succinic Acid | up |
|  | T | 5-Phosphonooxy-L-Lysine | up |
|  | L | Aminoadipic Acid | up |
|  | D | Glutaric Acid | up |
| Oxidative_phosphorylation | Z | Succinic Acid | up |
|  | gb | COX4 | up |
|  | cb | ATPeV0A, ATP6N | up |
| Tyrosine_metabolism | Z | Succinic Acid | up |
|  | na | frmA, ADH5, adhC | down |
| Pyruvate_metabolism | Z | Succinic Acid | up |
|  | na | frmA, ADH5, adhC | down |
|  | l | E1.1.1.40, maeB | up |
| Citrate_cycle__TCA_cycle_ | Z | Succinic Acid | up |
|  | h | OGDH, sucA | up |
| Fatty_acid_degradation | na | frmA, ADH5, adhC | down |
|  | D | Glutaric Acid | up |
