# Supplementary figures and images for: Comprehensive multi-omics and biochemical analysis to elucidate the molecular response mechanisms of gill and kidney tissues under acute salinity stress in Pseudobagras ussuriensis
Source: BMC Genomics. 2025 Jul 1;26:590. doi: 10.1186/s12864-025-11773-w (PMC12211720; doi:10.1186/s12864-025-11773-w)

**Supplementary Material 2**


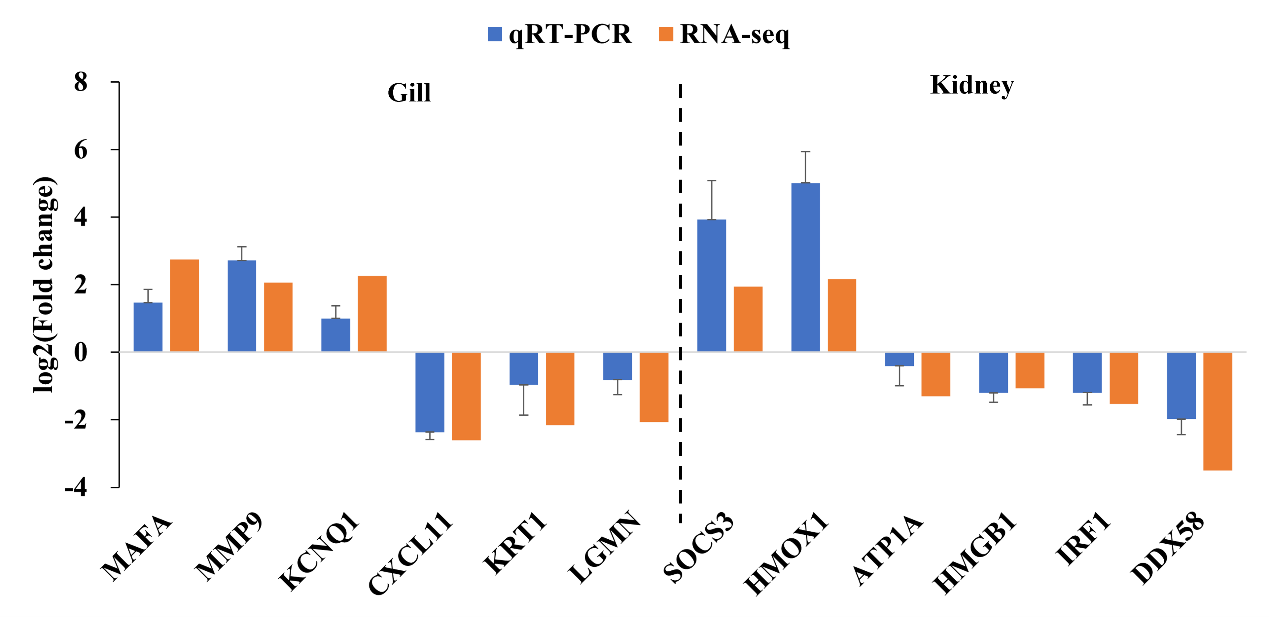


Figure 1. Comparison of gene expression data between RNA-Seq and qRT-PCR.

Supplement: Supplementary file 2 — Supplementary Material 2. [file 12864_2025_11773_MOESM2_ESM.docx]
